# Clinical information systems for the management of tuberculosis in primary health care

**DOI:** 10.1590/1518-8345.2238.2964

**Published:** 2017-12-11

**Authors:** Eliabe Rodrigues de Medeiros, Sandy Yasmine Bezerra e Silva, Cáthia Alessandra Varela Ataide, Erika Simone Galvão Pinto, Maria de Lourdes Costa da Silva, Tereza Cristina Scatena Villa

**Affiliations:** 1 Graduated, Graduated in Nursing, MSc, Nursing Department, Universidade Federal do Rio Grande do Norte, Natal, RN, Brazil, MSc in Nursing, MSc Scholarship holder of the Conselho Nacional de Desenvolvimento Científico e Tecnológico (CNPq).; 2 Graduated, Graduated in Nursing, MSc, Nursing Department, Universidade Federal do Rio Grande do Norte, Natal, RN, Brazil, MSc in Nursing.; 3 Especialist, Public Health Especialization, Residency, Medical Sciences Multicampi School, Universidade Federal do Rio Grande do Norte, Currais Novos, RN, Brazil, Residência Multiprofissional em Atenção Básica, Residency Scholarship funded by Ministério da Educação (MEC).; 4 Especialist, Doctor degree in Nursing, Adjunct Professor, Nursing Department, Universidade Federal do Rio Grande do Norte, Natal, RN, Brazil.; 5 PhD, Doctor degree in Health Sciences, Adjunct Professor, Nursing Department, Universidade Federal do Rio Grande do Norte, Natal, RN, Brazil.; 6 PhD, Doctor degree in Nursing, Full Professor, Maternal-Infant and Public Health Department at Escola de Enfermagem de Ribeirão Preto, Universidade de São Paulo, PAHO/WHO Collaborating Centre for Nursing Research Development, Brazil

**Keywords:** Descriptors: Tuberculosis, Information Systems, Health Information Management, Primary Health Care, Health Personnel, Delivery of Health Care, Integrated

## Abstract

**Objective:**

to analyze the clinical information systems used in the management of tuberculosis in Primary Health Care.

**Method:**

descriptive, quantitative cross-sectional study with 100 health professionals with data collected through a questionnaire to assess local institutional capacity for the model of attention to chronic conditions, as adapted for tuberculosis care. The analysis was performed through descriptive and inferential statistics.

**Results:**

Nurses and the Community Health Agents were classified as having fair capacity with a mean of 6.4 and 6.3, respectively. The city was classified as having fair capacity, with a mean of 6.0 and standard deviation of 1.5. Family Health Units had higher capacity than Basic Health Units and Mixed Units, although not statistically relevant. Clinical records and data on tuberculosis patients, items of the clinical information systems, had a higher classification than the other items, classified as having fair capacity, with a mean of 7.3 and standard deviation of 1.6, and the registry of TB patients had a mean of 6.6 and standard deviation of 2.0.

**Conclusion:**

clinical information systems are present in the city, mainly in clinical records and patient data, and they have the contribution of professionals linked with tuberculosis patients.

## Introduction

Tuberculosis (TB), an infectious disease considered a serious global public health problem, is a reflection of social and economic inequalities and has had major impacts on the morbimortality of the population, especially in the most vulnerable segments of developing countries^1^.

 This is reinforced by data from the World Health Organization showing that TB is among the ten leading causes of death in the world and that in 2015, 10 million people became ill and another 1.8 million people died due to this disease^2^. TB deserves special attention also because of the high number of people living in the world as carriers of the disease, but who are not notified and contribute to the dissemination and transmission in the community^3^.

Brazil ranks 18th in the absolute number of TB cases accounting for 0.9% of estimates in the world and 33% in the Americas. On average 70,000 new cases were diagnosed and approximately 4,400 deaths per year were reported from 2005 to 2014^4^.

As a way to reduce these indicators, the National Program for TB Control was created with the proposal of implementation of prevention, control and treatment actions for health surveillance of the TB cases in a horizontal and decentralized manner. Given this, Brazil has presented important data of reduction of incidence and mortality rates due to this disease^5^.

Its implementation is carried out mainly through the Primary Health Care (PHC), a complex and resolute strategy with the capacity to coordinate the Health Care Networks that, in this scenario, is organized through the Family Health Strategy (FHS). This strategy proposes the reorientation of the assistance model through the provision of teams that meet the needs of the population in the territory^6^.

For this, multiprofessional work and the creation of bond between these professionals and the population is necessary to allow the development of strategies of access, prevention and treatment of the disease. Such activities are possible when these professionals make use of the registration of important information about the health conditions of the users of the services^7^.

Clinical information systems in the health area are assistance instruments that allow the recording and analysis of data, so that professionals can relate health problems to their determinants, identify the risks of disease involvement, carry out the follow up, idealization and realization of prevention and treatment actions, so as to improve the quality of life of patients^8^.

Based on these findings and understanding the importance of the development of these systems in the scenarios of clinical performance, the question is: how are the clinical information systems used to treat TB in PHC?

To answer this question, the present study aims to analyze the clinical information systems used in the management of TB in PHC.

## Methods

A cross-sectional, descriptive and quantitative study was carried out with professionals from PHC units in a Brazilian northeast capital.

This study considered as Basic Health Unit (BHU), the one that provides care for spontaneous or programmatic demand of the population without the need for territory-based assigned registration of the clientele; as Family Health Units (FHU), the one in which there is delimitation of clientele and; Mixed Units (UM), the one that provides spontaneous or programmatic care in the basic specialties with hospitalization unit.

The research scenario was the city of Natal, capital of the State of Rio Grande do Norte, Brazil, and the study population consisted of 384 health professionals, namely: physicians, nurses, nursing technicians/assistants and community health agents (CHA). The sample was obtained through a sortition, with one professional per category from each unit, totaling 100 professionals. The values of P (Population relationship) = 0.5, CI (Confidence interval) = 95% and a sampling error of 5% were adopted. The inclusion criterion was having monitored cases of TB in PHC, and the exclusion criterion was being on health leave or vacations during the period of data collection. 

In case of refusal to participate in the study, other health professionals would be selected, provided they met the above criteria. Thirty-two professionals refused to participate in the research, being 17 physicians, one nurse, 12 nursing technicians/assistants and two CHA.

Data collection was authorized by the city’s health secretariat and took place between November 2013 and January 2014, in 27 PHC units of the city based on a structured questionnaire proposed by the *MacCooll Institute for Health Care Innovation.* This instrument was adapted and validated for the Brazilian reality for the evaluation of health professionals and local institutional capacity with the aim of developing a care model to chronic conditions adapted for the evaluation of TB control actions by the Group of Epidemiological and Operational Studies of the Brazilian Tuberculosis Research Network^9^.

This instrument is divided into seven dimensions and the present study used the one referring to the Clinical Information System, considered as a means of providing useful and timely information on users and on the populations affected by chronic diseases that use health services. It is also considered an aspect of effective care models that use population approaches^9^.

In this dimension, information about the capacity of the city, of the health units, of professionals and of the clinical information systems used for the management of TB patients were analyzed.

These items were classified according to their capacity to provide care, according to the following criteria: limited capacity (between 0 and 2), basic capacity (between 3 and 5), fair capacity (between 6 and 8) and optimal capacity (between 9 and 11).

Data were organized, categorized and codified in *Microsoft Office Excel spreadsheets* and exported to *IBM SPSS Statistics 20*, where they were statistically analyzed. Descriptive statistics were used for mean and standard deviation calculations of each item of the instrument. In order to compare the means between the types of health services or the function performed by health professionals in the unit, inferential statistics were used, through analysis of variance (ANOVA).

The research followed the ethical principles established in Resolution 466/2012 of the National Health Council, and was submitted and appreciated by the Research Ethics Committee of the Federal University of Rio Grande do Norte, receiving a favorable Opinion for its execution through the protocol number 456.332 and Certificate of Presentation for Ethical Appraisal 18675113.2.1001.5537.

## Results

 A total of 100 health professionals were interviewed, of which 22 (22%) were nursing technicians/assistants, 10 (10%) were physicians, 34 (34%) were nurses and 34 (34%) were CHA. The professionals were distributed in 27 health units, of which 71 (71%) were FHU, 27 (27%) to BHU and 02 (2%) to MU.

The professional categories, the healthcare units where the professionals work, and the items referring to the clinical information systems for the management of TB are presented in Table 1.

As for the role of each professional category in the use of clinical information systems, physicians and nursing technicians/assistants of the city presented basic capacity, while nurses and CHA showed a fair capacity with means of 6.4 and 6.3, respectively.

The clinical information systems had fair capacity in the city, with a mean of 6.0 and a standard deviation of 1.5. It was observed that 13 (48.1%) of the evaluated health units presented basic capacity and the other 14 (51.9%), fair capacity.

The data show that there was a difference in the capacity among the types of health units, although without statistical significance in the ANOVA. The MU were classified as having basic capacity, and the FHU and BHU as having a fair capacity.

**Table 1:** Characterization of the monitoring of clinical information systems of tuberculosis in Health Units, Natal, RN, Brazil, 2013-2014

**Category analyzed**	M*	**SD** ^**†**^	**N** ^**‡**^	**Classification**
**Function of Professionals in the Unit**				
**Community Health Agent**	**6.2**	**1.3**	**34**	**Fair**
**Nurse**	**6.4**	**1.6**	**34**	**Fair**
**Physician**	**5.4**	**1.81**	**10**	**Basic**
**Nursing technicians/assistants**	**5.7**	**1.7**	**22**	**Basic**
**City**	**6.0**	**1.5**	**100**	**Fair**
**Health Unit**				
**Basic Health Unit A**	**4.2**	**0.24**	**2**	**Basic**
**Basic Health Unit B**	**6.3**	**1.0**	**4**	**Fair**
**Basic Health Unit C**	**5.3**	**0.35**	**2**	**Basic**
**Basic Health Unit D**	**5.8**	**1.7**	**3**	**Basic**
**Basic Health Unit E**	**5.1**	**1.1**	**2**	**Basic**
**Basic Health Unit F**	**6.0**	**1.7**	**5**	**Fair**
**Basic Health Unit G**	**4.9**	**0.12**	**2**	**Basic**
**Basic Health Unit H**	**5.3**	**1.2**	**2**	**Basic**
**Mixed Unit A**	**4.7**	**2.1**	**2**	**Basic**
**Family Health Unit A**	**7.8**	**0.59**	**4**	**Fair**
**Family Health Unit B**	**6.2**	**1.6**	**7**	**Fair**
**Family Health Unit C**	**5.4**	**1.2**	**9**	**Basic**
**Family Health Unit D**	**7.1**	**2.3**	**3**	**Fair**
**Family Health Unit E**	**7.8**	**0.77**	**3**	**Fair**
**Family Health Unit F**	**4.0**	**0.33**	**3**	**Basic**
**Family Health Unit G**	**8.0**	**0.76**	**3**	**Fair**
**Family Health Unit H**	**6.3**	**1.3**	**2**	**Fair**
**Family Health Unit I**	**6.2**	**1.3**	**10**	**Fair**
**Family Health Unit J**	**7.5**	**0.58**	**3**	**Fair**
**Family Health Unit K**	**5.2**	**1.5**	**4**	**Basic**
**Family Health Unit L**	**7.3**	**1.3**	**4**	**Fair**
**Family Health Unit M**	**5.7**	**0.94**	**2**	**Basic**
**Family Health Unit N**	**6.1**	**3.3**	**3**	**Fair**
**Family Health Unit O**	**8.2**	**2.0**	**3**	**Fair**
**Family Health Unit P**	**5.5**	**1.0**	**4**	**Basic**
**Family Health Unit Q**	**5.1**	**1.1**	**4**	**Basic**
**Family Health Unit R**	**6.1**	**0.67**	**5**	**Fair**
**Type of Health Service**				
**Basic Health Units** ^**§**^	**6.0**	**1.5**	**27**	**Fair**
**Family Health Units** ^**§**^	**6.2**	**1.6**	**71**	**Fair**
**Mixed Unit** ^**§**^	**4.7**	**2.1**	**2**	**Basic**

*M: mean; †SD: standard deviation; ‡N: number; §Indicates lack of statistical significance in the analysis of variance.

Regarding the items that make up the clinical information system, shown in Table 2, it was observed that they presented fair capacity in the case of the following items: clinical record (mean of 7.3 and standard deviation of 1.6) and registry of TB patients (mean of 6.6 and standard deviation of 2.0).

**Table 2:** Characterization of the items that make up the clinical information systems of tuberculosis in the Health Units, Natal, RN, Brazil, 2013-2014

**Items of Clinical Information Systems**	M*	**SD** ^**†**^	**N** ^**‡**^	**Classification**
**Clinical record**	**7.3**	**1.6**	**100**	**Fair**
**Registry of tuberculosis carriers**	**6.6**	**2.0**	**100**	**Fair**
**Notices and reminders for health professionals issued by the Epidemiological Surveillance, laboratories, among others**	**5.6**	**3.2**	**100**	**Basic**
**Feedback**	**5.1**	**3.1**	**100**	**Basic**
**Information on tuberculosis patients at risk of abandonment of treatment, failure and death**	**5.8**	**2.8**	**100**	**Basic**
**Care plan for people with tuberculosis**	**5.8**	**1.5**	**100**	**Basic**

*M: mean; †SD: standard deviation; ‡N: number

Therefore, the record is always available to professionals in the unit and includes clinical data, and diagnostic and therapeutic records. These documents also have information on return scheduling, supervised or self-administered treatment, request and results of control screenings, and contact surveillance.

TB carrier records contain information on at least three types of forms. This cases include identification data such as telephone, address and other social characterizations, allowing a description of the users assisted according to extracts. Such information may be present or come from TB patient registry books and follow-up of treatment, notification or investigation form, and Directly Observed Treatment (DOT) record sheets.

The remaining items had basic capacity. The fact that these professionals use this classification regarding the receipt of notices and reminders to health professionals issued by the Epidemiological Surveillance, laboratories and other services, shows that, for them, the responsibility of monitoring is attributed to another type of health service, as referral outpatient clinics, hospitals, and epidemiological surveillance, for example.

With regard to the feedback for the performance of the HU in TB control, these data are generally disclosed once a year and consist in the number of cases and sputum smear examinations.

Two other items were also classified as having basic capacity. One of them corresponds to information about TB patients at risk of abandonment, failure and death. These data are available, but access is limited to local staff. In addition, the care plan for TB patients was also classified as having basic capacity. This includes the prescription of medications, requests for tests, and general nursing guidelines.

## Discussion

The fair capacity attributed by nurses and CHA to the clinical information systems calls attention to their key role in the follow-up of TB carriers. This was observed in a study that showed that, although clinical information systems are tools of paramount importance in the team’s performance, their use is centered on the figure of the nurse. It was also observed that CHA are strategic components to intermediate the actions of the team toward users under treatment^10^.

This same capacity was also observed in more than half of the analyzed health units and made the city also to be classified as having fair capacity. It should be emphasized that this result may have an influence on the fact that the city has its HCN network almost exclusively constituted by FHU. 

Among the items that compose the clinical information systems of TB, it is observed that the clinical records were classified as fair. It is worth noting that, during the period of data collection, the city did not yet have a computerized medical record, which may have interfered with the clinical management of TB.

The medical record is a document of paramount importance to communicate information about each patient within the team, making it possible to document all the activities performed by professionals in the treatment of TB carriers. Therefore, it is important to keep the medical record updated and correctly filled. This will help to follow the evolution of the treatment of patients. The adequate filling of the records is a responsibility of the whole team^11^.

The registry of TB patients also presented a fair classification. The importance of this registry for the TB control program comes from the fact that the information filled in the notification form for diagnosed cases of TB and in the treatment control book are the ones that are sent monthly to the National Disease Notification System (SINAN). 

A study conducted nationwide in 2016 showed that 43.6% of the health units did not have a registry of TB patients and 49% of these units did not know or did not answer the question regarding the follow-up registry of TB cases^12^. 

Research demonstrated the importance of using the registries of the profile of TB carriers to favor a better therapeutic adherence. This is possible through the systematization of services by the provision of DOT actions, TB guidelines, incentives to conduct tests, program improvement, among others^13^.

The fact that, in the present study, notices and reminders to health professionals about the occurrence of new cases had a basic character points to the difficulty in guiding TB control actions, because the lack of knowledge of the health situation of assigned territories make it impossible to create successful strategies^14^.

Regarding the basic character of the feedback of information, a study found similar results^9^. It is obvious that this aspect detected in the study make it difficult to plan interventions, as health professionals are unable to monitor and evaluate the control actions. Consequently, the health team becomes only the recipient of information and not a participant in the process of providing care for TB patients^15^.

Regarding the basic classification of the item information about TB patients at risk of abandonment, failure and death, this situation suggests the need for greater attention on the part of professionals, because, as observed in the study, neglect of information on treatment abandonment and other situations are a reson of concern for the control of TB^16^.

One of the main obstacles to the control of TB is the abandonment of treatment; it has an impact both on the increase of the cost of treatment, and on the mortality and relapse rates. It is also observed that this lack of adherence may increase the spread of the bacillus, and also its resistance. This is especially true among young people with low schooling, alcoholics and people with mental illness. Strategies to encourage treatment adherence among this publics are necessary^17^.

The care plan for TB patients presented in this study was classified as basic, including prescription of medications, requests for tests, and general nursing guidelines.

Among the strategies that can contribute to the implementation of the care plan are the adoption of measures that are attractive and easily accessible to users. An example of this is the adoption of mobile applications that, with the increase in the use of smartphones, can be excellent strategies in the creation of bond and can contribute to the continuity of treatment of these patients^18^.

Therefore, it is indispensable to organize the care technologies necessary for the health care of TB patients. This organization has elements that can strengthen the bond between professionals and patients, and the health team itself. This is because the internal organization of work in the health unit, through the management of TB control policies and programs by all those involved in the management of the case, enables the dissemination of knowledge about the patient, and the generation of relevant and indispensable information to the reference system, when necessary^9^.

As a way to expand this care and obtain better results regarding the availability of information on the treatment of TB carriers in PHC, the constant training of professionals and access to quality information materials, as pointed out in the study, it is recomendable^19^. The continuous stress of the important roles of these professionals in the control of TB is also one of the ways to achieve the effective dissemination of information on TB treatment.

Regarding the limitations to the accomplishment of this study, we highlight the difference in the number of professionals in the professional categories, which made data collection difficult, and the inferences to the population, through these categories, impossible.

## Conclusion

The clinical information systems in the city presented fair capacity, and nurses and CHA were the professionals that contributed the most to these results. On the other hand, physicians and nursing technicians/assistants had basic capacity in the use of these systems for the clinical management of TB.

The data showed that FHU were presented better classification when compared to the others units, which is mainly due to the fact this units offer a care that allows a greater bond between professionals and users, which in turn contribute to the better use of the information.

Clinical records and registry of TB patients are the items that contributed the most to the clinical information systems of the disease in the city studied, presenting a fair capacity. On the other hand, there was little influence of the notices and reminders for health professionals issued by the Epidemiological Surveillance, laboratories, among others; feedback of information; information about TB patients at risk of abandonment, failure and death; and care plan for TB patients. All of these items presented basic capacity. 
